# Numerical simulation of red blood cells migration and platelets margination for blood flow in micro-vessels with fusiform aneurysms

**DOI:** 10.1038/s41598-025-22429-w

**Published:** 2025-10-27

**Authors:** Ahmed Elhanafy, Samir Elsagheer, Sameh Nada

**Affiliations:** 1 Mathematics and Engineering Physics Department, Faculty of Engineering, Mansoura University, Mansoura, 3551 Egypt; 2 Computer Science and Information Technology Programs, Egypt-Japan University of Science and Technology (E-JUST), P.O. Box 179, New Borg El-Arab City, 21934 Alexandria Egypt; 3 Faculty of Engineering, Aswan University, Aswan, 81528 Egypt; 4 Department of Energy Resources Engineering, Egypt-Japan University of Science and Technology (E-JUST), P.O. Box 179, New Borg El-Arab City, 21934 Alexandria Egypt; 5 Mechanical Engineering Department, Benha Faculty of Engineering, Benha University, Benha, 13512 Qalubia Egypt

**Keywords:** Fusiform aneurysms, RBC migration, Platelet margination, CFL thickness, Hematocrit variation, Biophysics, Cardiology, Computational biology and bioinformatics, Diseases, Engineering, Mathematics and computing

## Abstract

Understanding several micro-vascular diseases depends mainly on examining the dynamic behavior of blood cells, especially the red blood cells (RBCs) and platelets. For instance, the dynamics of RBCs and platelets are significantly impacted by micro-vascular diseases such as aneurysms, which may lead to many disorders. The oxygenation process, for example, depends on the motion and velocity of the RBCs. Important hemodynamic parameters such as the wall shear stress (WSS) and the cell-free layer (CFL) thickness are affected by the motion of the RBCs and platelets. Thus, the main objective of the current study is to introduce more insights into cellular blood dynamics in micro-vessels with fusiform aneurysms, which have important clinical implications, by examining some important hemodynamic parameters. This study examines the migration of RBCs and their velocities under different hematocrit levels. Furthermore, the effect of hematocrit variation on platelets’ margination, the CFL, and the WSS is investigated. The simulations are performed using a validated code developed cellular flow simulations. The obtained results show that decreasing the hematocrit value increases the proportion of migrated RBCs, and hence the CFL thickness increases, which significantly affects blood apparent viscosity, especially at the aneurysm zone, and hence affects the local WSS and the endothelial cell in the vessel wall tissue. In addition, it is found that the fusiform aneurysm reduces the velocity of the RBCs by more than 83%, which can affect the oxygenation process. The obtained results exhibit an asymmetrical trend up and downstream of the aneurysm zone, with a thinner CFL at the divergent part of the aneurysm. These results are beneficial for medical microfluidic devices as well as for understanding many microvascular diseases.

**Supplementary Information:**

The online version contains supplementary material available at 10.1038/s41598-025-22429-w.

## Introduction

 Blood is a complex fluid that consists of several components; however, the main components are RBCs, white blood cells (WBCs), platelets, and plasma^1–3^. RBCs or erythrocytes are nuclei-free cells which give blood its characteristic red color. They have many crucial roles in the human body^4^. Healthy RBCs live for about 120 days; after this time, the cells are worn out and damaged^5^. Nearly all basic physiological processes are dependent on the dynamics of the RBCs^6^. The most important role of the RBCs is to transport oxygen from the lungs and essential nutrients to all the tissues of the human body. The biconcave shape with the elasticity of the membrane helps the RBCs to do this vital role even in very small capillaries where the RBCs can be squeezed and pass through the narrow vessels one by one^7^. Another important role of the RBCs is to transport metabolic byproducts such as carbon dioxide from the tissues back to the lungs. The dynamic behavior of the RBCs is crucial, and their analysis is important to understand several diseases such as diabetes mellitus, malaria, and sickle cell anemia^8^. The second important components of blood are platelets, which are released from megakaryocytes. Platelets exist in blood circulation for about 5–7 days. The primary function is to regulate hemostasis and thrombosis^9^. In addition to the vital role of the platelets in preventing excessive bleeding during injuries, platelets have a great impact on the integrity of the endothelial cells of the vessel wall. Plasma mainly consists of water, with approximately 55% of blood volume. The RBCs approximately represent the remaining volume fraction^10,11^. The volume of the WBCs and the platelets is very small, and therefore, it can be neglected. Therefore, studying the dynamics of RBCs in platelets in diseased micro-vessels is important to understand such diseases.

One of the common diseases in micro-vessels is micro-aneurysms. Micro-aneurysms are dilations of the capillaries that are commonly found in the retinal capillaries of the diabetic eye^12^. According to morphology, micro-aneurysms can be classified into three categories: saccular, fusiform, and focal bulges. In the saccular aneurysm, the dilation is asymmetric around the axis of the vessel, whereas it is symmetric in the fusiform aneurysm^12^. Retinal micro-aneurysm formation is considered one of the earliest clinical indicators of diabetic retinopathy, which may lead to subsequent retinal edema and vision loss^13^. Several hemodynamic factors, such as the WSS, have a significant role in the initiation and rupture of micro-aneurysms^14^. There is strong evidence that the WSS is strongly linked to the thickness of the CFL by lowering the effective blood viscosity^15^. Therefore, examining the dynamics of cellular blood flow in micro-vessels with micro-aneurysms and investigating the effect of the hematocrit level on the WSS, the CFL thickness, and plasma flow velocity are crucial due to their direct effect on the integrity of the vessel wall and the possibility of the aneurysm rupture.

Several studies have been conducted to introduce three-dimensional simulations for cellular blood flow in micro-vessels. Hyakutake and Nagai^16^ Constructed a 3D microvascular bifurcation model to investigate the effect of the bifurcation angle and shape on the distribution of the RBCs. They found that the bifurcation angle and the diameter of the daughter vessel have a remarkable effect on the distribution of the RBCs. Ye et al.^17^ performed three-dimensional numerical simulations of RBCs in a microvascular network. They found that decreased deformability and increased aggregation strength have a similar effect on the RBC distribution. Li et al.^18^ conducted a study on cell suspension flow in complex micro-networks. Their results were compared with previously published numerical results, with good agreements were observed on the velocity field, cell deformation, and rheological behaviors of cells. Wang et al.^19^ performed a numerical simulation of the Margination and adhesion dynamics of tumor cells in a real microvascular network. Similarly, Balogh and Bagchi^20^ presented a numerical simulation of the flow of the RBCs in 3D microvascular networks to resolve the dynamics and the deformation of the individual cells. Reduction of hematocrits and flow rates is observed in the daughter and mother vessels due to cells jamming at the vascular bifurcation. Li et al.^21^ analyzed and predicted the hematocrit distribution in complex microvascular networks. Their results revealed that the hematocrit is strongly linked to the complexity of the network and the size of the vessel. Ye and Peng^22^ performed a numerical simulation to study the deformation and the aggregation of multiple RBCs in a micro-vessel with a bifurcation. Their results revealed that the leading RBC had more deformation compared with other RBCs. Another study was performed by Ye et al.^23^ to examine the motion and deformation of the RBCs in rectangular microchannels. Xiao et al.^24^ studied the effect of the aggregation of the RBCs in stenosed micro-vessels. Their results indicated that the aggregation of the RBCs had occurred at the proximal end of the stenosis. As a result, a thinner CFL was formulated. In the same context, Zhao and Xie^25^ performed a numerical simulation of blood flow in stenosed micro-vessels using a two-fluid model. They found that the inlet velocity and the hematocrit level had a remarkable impact on the CFL thickness. For straight vessels, Fedosov et al.^26^ simulated the flow of the RBCs and predicted the thickness of the CFL for different hematocrit levels and the vessel diameters. The results are consistent with other experimental works. Chang et al.^27^ simulated the flow of the RBCs and platelets in cylindrical vessels by considering different cell shapes, sizes, and RBC deformability. They estimated the CFL thickness for different flow conditions.

Few studies have been found in the literature to examine the cellular blood flow in micro-vessels with aneurysms. Elhanafy et al.^28^ investigated the effect of curvature degree as well as the hematocrit on the dynamics of the RBCs and other hemodynamic parameters for cellular blood in curved micro-vessels with saccular aneurysms. They found that the CFL thickness is highly dependent on the curvature degree, especially at the apex of the curved vessel. Czaja et al.^29^ investigated the effect of stiffened diabetic RBCs on the WSS in a micro-aneurysm. Their results revealed that the average WSS and WSS gradients increased with the increase in stiffness of the RBCs. Wu et al.^30^ presented a numerical simulation of blood flow in two aneurysmal vessels using a multi-component approach to examine platelet transport. The proposed model accurately predicted the enrichment of the platelets near the wall. Furthermore, the obtained results indicated that the concentration of the platelets inside the aneurysm increased with the increase in the Reynolds number. Similarly, Li et al.^31^ presented a computational investigation of the transport of blood cells in retinal micro-aneurysms. The obtained results predicted increased blood velocity and hematocrit in the parent vessel of the micro-aneurysm. Cai et al.^32^ provided a new model to determine the velocity, pressure, and stress fields of blood in microcirculations using sequential images from microfluidics experiments and machine learning. They considered the saccular aneurysms of different sizes.

According to the authors’ best knowledge, the cellular blood flow in micro-vessels with fusiform aneurysms has not been sufficiently examined. While the previous studies considered the cellular blood flow in straight vessels or vessels with saccular aneurysms, the micro-vessels with a fusiform aneurysm type are not considered, where the dilation affects the dynamics of the main stream flowing cells. Additionally, some important features, such as the CFL thickness, platelet margination, and the migration of the RBCs, are not fully understood. Thus, the main objective of the current study is to examine the cellular blood flow dynamics in the fusiform aneurysm under the variation of the hematocrit level. In the current study, the RBCs’ migration, platelet transport, and CFL thickness are examined under different flow conditions. The effect of the aneurysm zone on the velocity of the RBCs is also investigated. Furthermore, the plasma flow velocity and the WSS are considered.

## Physical model

**Fig. 1:**
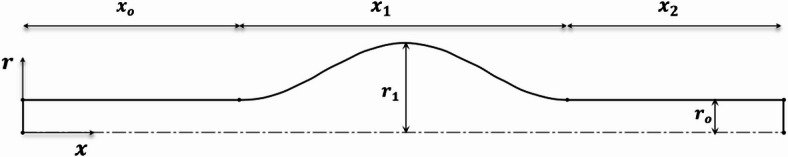
The variation of the vessel radius with the axial direction.

Figure 1 presents the variation of the diameter along a micro-vessel with a fusiform aneurysm. The dilation of the vessel wall is mathematically described by a sine function as follows^33^:

1 $$r^{*} = \left\{ {\begin{array}{*{20}l} {\frac{7}{{16}}~\left[ {1 + sin~\left( {2\pi \frac{{x - x_{o} }}{{5D}} - \frac{\pi }{2}} \right)} \right] + 0.5,} \hfill & {x_{o} \le x \le x_{o} + x_{1} } \hfill \\ {0.5} \hfill & {otherwise,} \hfill \\ \end{array} } \right.$$

where *r* is the radial coordinate system of the vessel; ${r_o}$ is the normal radius of the vessel; *D* is the diameter of the vessel; ${r_1}$ is the maximum radius of the fusiform aneurysm and ${r^*}$ is the non-dimensional radial distance defined by ${r^*}=\frac{r}{D}$. In this study, the normal diameter of the vessel is 30 $\mu m$, whereas the maximum diameter at the center of the aneurysm is 82.54 $\mu m$. The total vessel length is 350 $\mu m$.

## Mathematical models and numerical methods

For cellular blood flow modelling, the RBCs are considered elastic membranes immersed in the surrounding plasma, considering the interaction between the plasma forces and the deformation of the RBCs. The surrounding plasma is an incompressible Newtonian fluid with a constant viscosity^34^ and the platelets are considered weakly deformed particles^35^. The arterial wall is assumed to be rigid, and the pulsatile nature of blood flow in micro-vessels is neglected. For the flow solver, a validated open-source code developed for cellular blood flow simulation, HemoCell, is used in the current work^36^. Several computational works in the literature have confirmed the accuracy and validity of the proposed code^37–43^.

### The red blood cell and platelet mechanical models

The RBC membrane is considered a viscoelastic material and consists of three layers: a phospholipid bilayer and a cytoskeletal network^44^. This structure provides the RBC with its elasticity and allows it to pass through narrow vessels^10,45^. As a result, the RBC’s mechanical behavior depends on the elastic membrane’s structure. In HemoCell, a discrete spring network model is adopted to represent the cytoskeletal network using a triangular mesh. In the suggested model, four different types of forces are considered^42^. The proposed forces are applied to the triangular mesh vertices, which define membrane deformation and its interaction with plasma forces. The mathematical formulation of the applied forces is briefly described in Appendix I. For platelet modelling, it is considered a weakly deformed particle with approximately an ellipsoidal shape. The mechanical model used for platelet modeling is a simplified version of the model described for the RBCs. The key factor that detects the mechanical behavior of both types is the chosen values of the free parameters^46^. The numerical values of the free parameters for both the RBCs and platelets are introduced in Appendix II, Table AII. 2.

### Fluid-membrane interaction (LBM-IBM)

In HemoCell, the LBM is proposed to simulate the flow of plasma. As the RBCs are considered moving boundaries immersed in the surrounding fluid, the IBM coupled with the mechanical model is considered to model the deformation of the RBCs due to plasma forces. A brief description of both the LBM and the IBM is introduced in Appendix III and Appendix IV, respectively.

### Computational setup

In this study, the physical domain 350 $\mu m$x30$~\mu m$ × 30$~\mu m$ is used. In the LBM, it is important to create a link between the physical domain and the lattice domain, and this link is performed by satisfying both geometrical and dynamical similarities. The first step is to convert the physical equations into a non-dimensional form, where the only non-dimensional parameter, in our case, the Reynolds number, arises. To satisfy dynamic similarities between the physical domain and the lattice domain, unit conversion is required. The Reynolds number ($Re=\frac{{UL}}{\nu }$) has to be matched with the lattice Reynolds number ($R{e_l}=\frac{{{U_l}M}}{{{\nu _l}}}$) where ${U_l}$ is the inlet lattice velocity, ${\nu _l}$ is the lattice viscosity, and *M* is the number of lattices in the reference length. Hence, ${U_l}$ and ${\nu _l}$ should be chosen to satisfy the stability of the LBM. In HemoCell, for pipe flow, it is recommended to use a lattice reference length two times the physical reference length, i.e., the lattice unit $\Delta x=0.5 \times {10^{ - 7}}\mu m$. Therefore, the corresponding lattice domain will be 700 × 60 × 60 lattices. The ${\nu _l}$ can be calculated to satisfy the dynamical similarities by using a small lattice inlet velocity (less than 0.1) to ensure the incompressibility, and the lattice viscosity should be related to the relaxation time via the relation ${\nu _l}=\left( {\tau - 1/2} \right){c_s}^{2}\Delta t$ where the relaxation time should be greater than 0.5.

To run a simulation case in HemoCell, certain parameters need to be defined using a configuration file. In this file, the diameter of the vessel, the number of lattices in the reference direction, the Reynolds number, and the time step are defined. The parameters of the mechanical model of the RBCs and platelets are also included. In the present study, a Dell workstation with 2 Intel Xeon(R) Gold 6230R 64-bit CPUS with 104 Cores is used. The Linux operating system, Ubuntu 20.04.6 LTS, is installed to run HemoCell code. We used 100 cores for all the simulation cases where parallel computing is performed based on this number of cores.

### Initial and boundary conditions

**Fig. 2:**
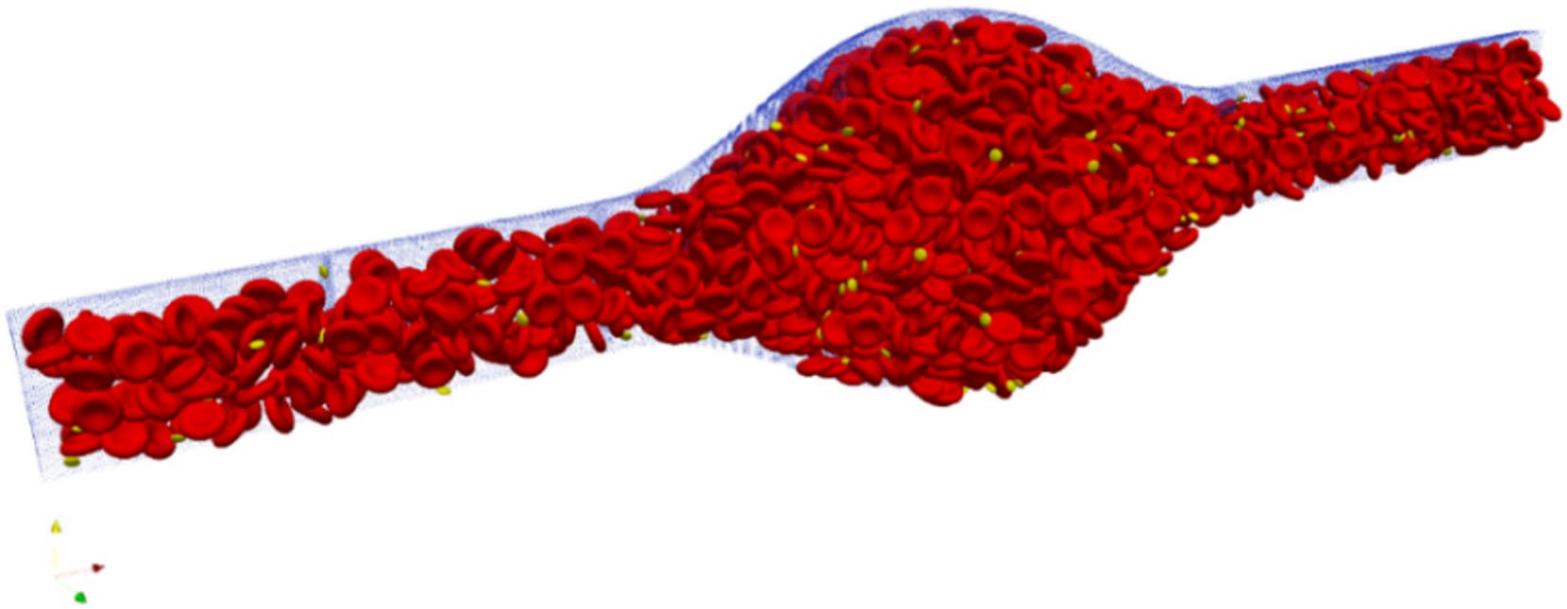
The initial positions of undeformed cells in a micro-vessel with a fusiform aneurysm for a hematocrit of 45% (generated by HemoCell v2.3 (https://www.hemocell.eu/) and the software Paraview v5.1.1(https://www.paraview.org/)).

An important issue related to cellular blood flow simulation is choosing the initial positions for blood cells. An example of the initial positions used in this study is shown in Fig. 2. The figure is generated using the open-source software, HemoCell v2.3^47^, and the software Paraview v5.1.1^48^. In HemoCell, packing cell software is provided to generate the initial positions using a script to determine the position and orientation of each cell. The position and the orientation of each cell are stored in a POS file, which can be easily implemented in the simulation^36^. In the packing cell software, it is required to determine the domain size in lattice units. In addition, it is required to input the desired hematocrits and the required ratio of platelets, and then the software calculates the required RBCs and platelets based on the input data. The Packing Cell Solver uses algorithms to populate the vessel with RBCs based on the given hematocrit and ensures there is no overlap between cells.

Periodic boundary conditions are applied at the inlet and the outlet of the vessel. At the wall of the vessel, bounce-back boundary conditions are applied^7^.

## Validation

HemoCell is a validated code on different levels of simulations from single-cell validation to the validation of blood flow with a highly dense suspension of cells. Several studies have used the HemoCell code for cellular blood flow simulations and have compared their results with other experimental and computational works with an acceptable degree of agreement^28,36^.

## Results and discussion

The cellular blood flow in micro-vessels with a fusiform is examined under different values of hematocrit. The migration of the RBCs and the platelet margination process are considered, especially in the aneurysm region. Other hemodynamics parameters, such as the velocity profiles and the WSS, are also considered. Owing to the nature of cellular blood flow in micro-vessels, the Reynolds number is typically low, and hence the value of the Reynolds number in this study is 0.5, as suggested by Hsieh^49^ and Czaja et al.^50^. To clarify the effect of hematocrit variation on cell dynamics and plasma flow, different values are considered, namely, 10%, 20%, 30%, and 45%. The number of RBCs and platelets for each case is summarized in Table 1.

**Table 1:** The number of RBCs and platelets for different hematocrits.

Case	Number of RBCs	Number of platelets
hematocrit 45%	1627	194
hematocrit 30%	1155	141
hematocrit 20%	742	88
hematocrit 10%	368	39

### Red blood cell migration

**Fig. 3:**
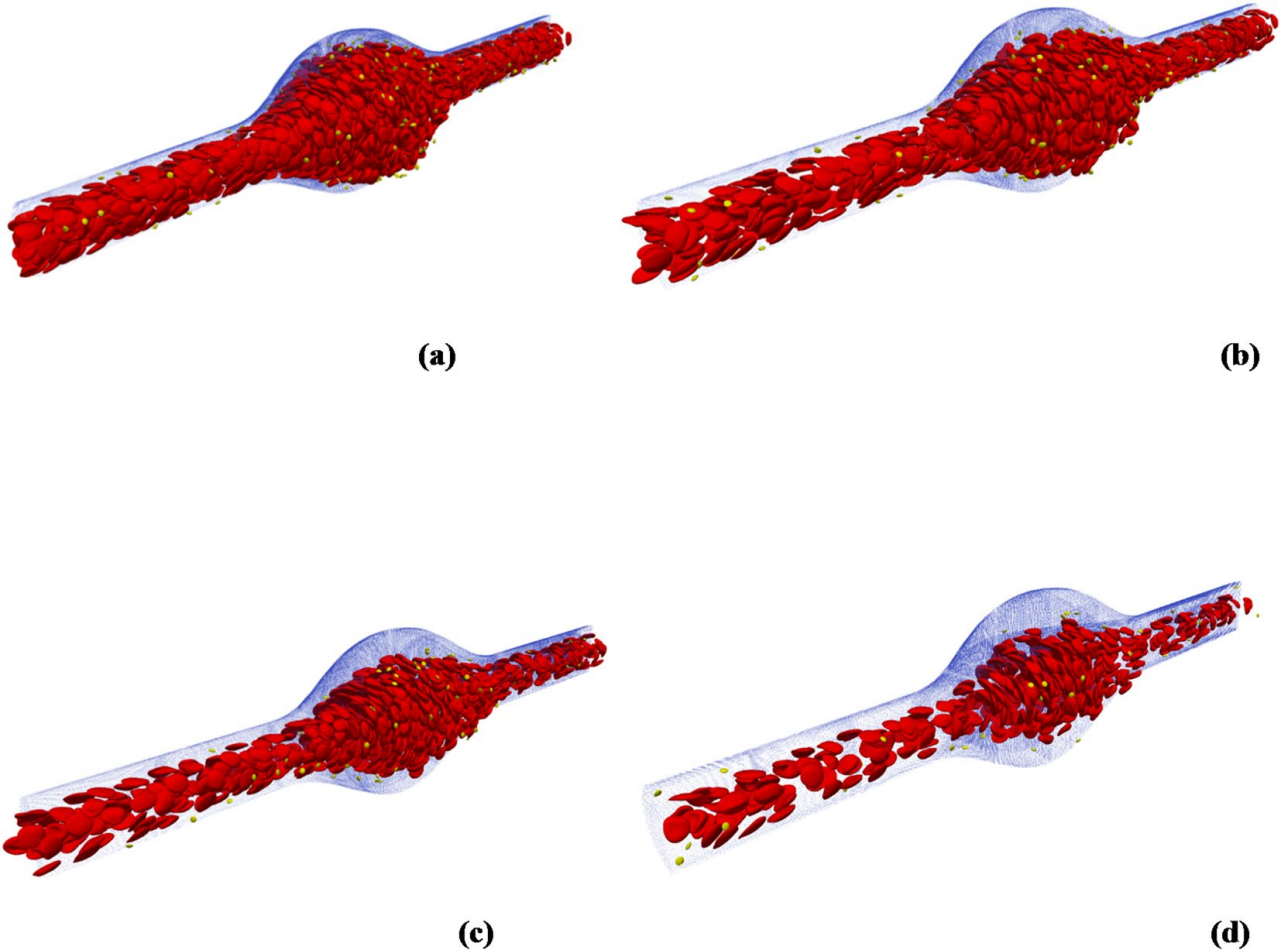
Snapshots of the flowing blood cells for different hematocrits: (a) 45%, (b) 30%, (c) 20% and (d) 10% ((generated by HemoCell v2.3 (https://www.hemocell.eu/) and the software Paraview v5.1.1(https://www.paraview.org/)).

One of the most prominent features of cellular blood flow dynamics is the migration of RBCs. In this phenomenon, the RBCs move towards the centerline of the vessel. As a result, a CFL is formed near the vessel wall, which is known as the Fahraeus-Lindqvist layer^51,52^. Furthermore, the RBC migration induces stiffer platelet margination by volume exclusion^53^. Figure 3 shows the distribution of the blood cells through the micro-vessel for different hematocrits. The snapshots are generated using the HemoCell v2.3^47^ at the end of the simulation.

**Fig. 4:**
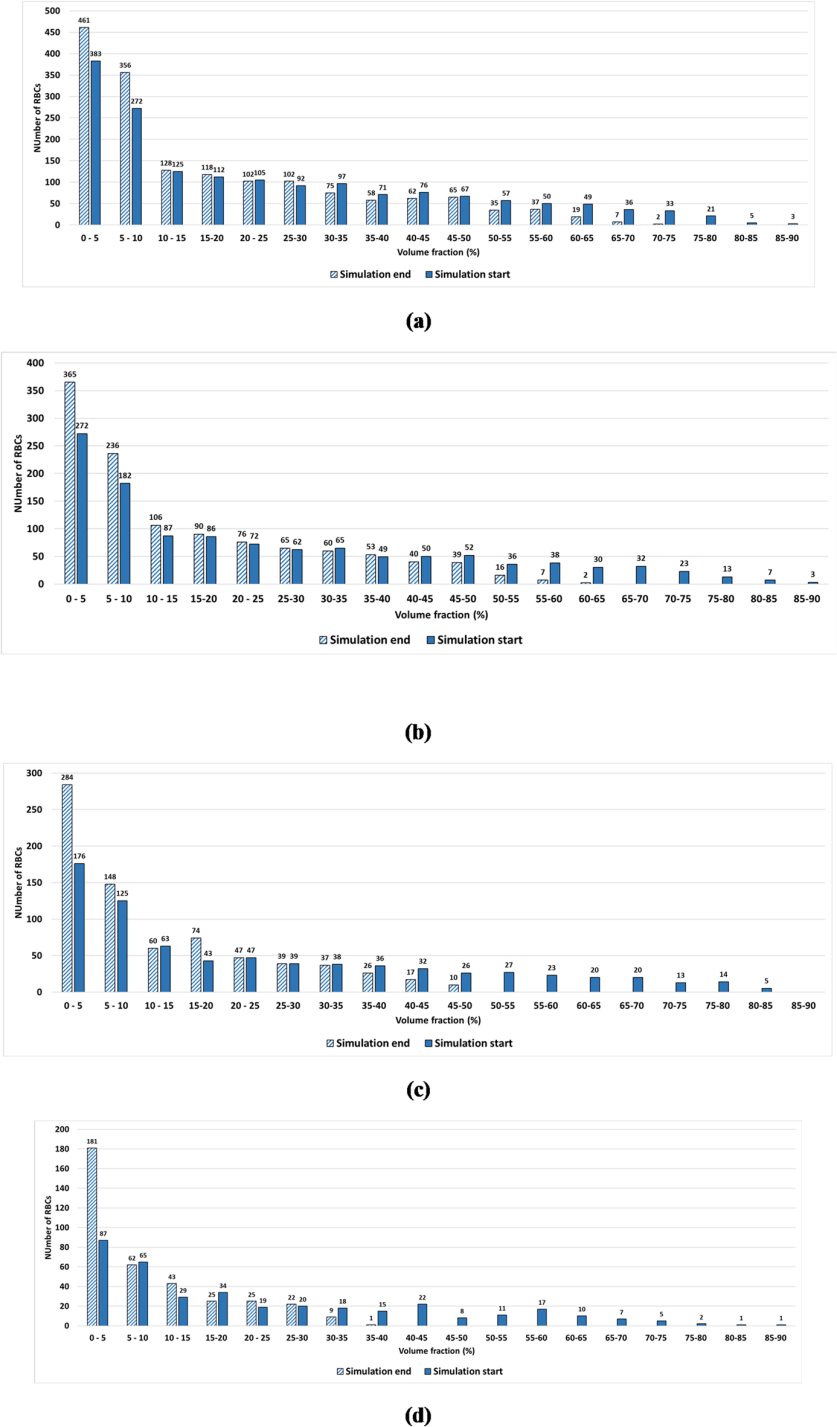
The RBCs migration: radial distribution of the RBCs for different hematocrit levels: (a) 45%, (b) 30%, (c) 20%, and (d) 10%.

**Fig. 5:**
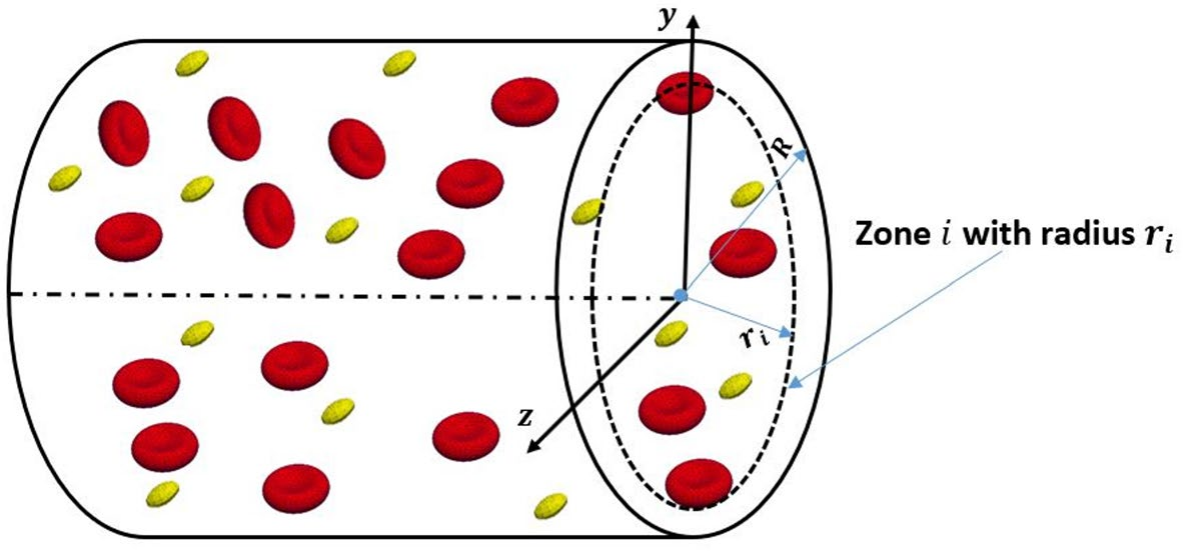
A schematic diagram for dividing the vessel volume into several zones (generated by HemoCell v2.3 (https://www.hemocell.eu/) and the software Paraview v5.1.1(https://www.paraview.org/)).

To quantify the migration of the RBCs, histograms for the distribution of the RBCs are presented for different values of the hematocrits. The obtained results are compared with those obtained at the start of the simulation in different regions of the vessel. Figure 4 presents the variation of the number of concentrated RBCs with the vessel volume fraction for different hematocrits. The volume fractions are obtained by normalizing the vessel volume with a constant volume based on the maximum diameter of the aneurysm. The distributions of the RBCs and the platelets are obtained for different volume zones along the radial direction from the center of the vessel to the arterial wall. Accordingly, the vessel volume is divided into 10 zones or layers to clarify the migration and margination processes. Each zone represents a volume fraction of the volume of the vessel, as indicated in the schematic figure, Fig. 5.

The migration of the RBCs is confirmed for all the examined cases, particularly with a hematocrit of 10% which proves the validity of the proposed code. At a hematocrit of 45%, the number of concentrated RBCs in a volume fraction of 10% is 817, which represents about 50.2% of the total RBCs. Comparing the results with an initial distribution of the RBCs, it is found that about 9.95% of the total number of RBCs migrated towards the centerline of the vessel. At a hematocrit of 10%, the proportion of the migrated RBCs is 24 0.72%, whereas it reached 12.72% at a hematocrit of 30%.

From the previous results, it is found that the maximum proportion of the migrated RBCs occurs at a hematocrit level of 10%. This is due to the reduction in the flow resistance, which depends on the presence of RBCs. Another key factor that induces the RBC migration is the nature of the fusiform aneurysm with its divergent part, which reduces the aggregation of the RBCs. In contrast, in the stenosed vessel cases, it is found that the presence of the stenosis increases the accumulation of the RBCs, and hence decreases the migration towards the center of the vessel and decreases the CFL thickness, as reported by Xiao et al.^24^.

### Red blood cell velocity distribution

**Fig. 6:**
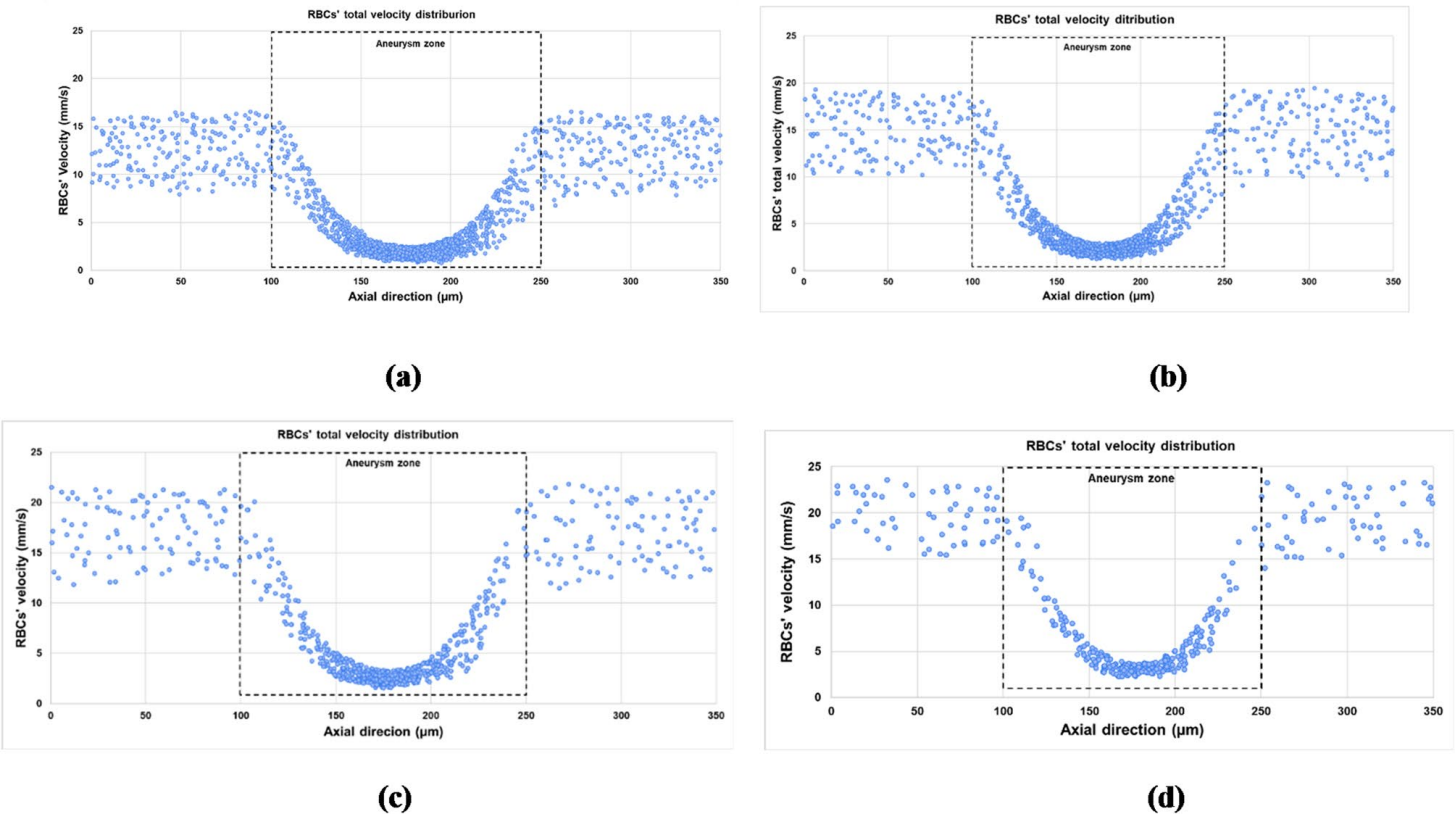
The effect of hematocrit variation on the RBC total velocity distribution in the fusiform aneurysm: (a) hematocrit 45%, (b) hematocrit 30%, (c) hematocrit 20% and (d) hematocrit 10%.

As the oxygenation process is highly affected by the velocity of the RBCs, it is convenient to investigate the effect of hematocrit variation on the velocity of the RBCs in different regions in the vessel. For hematocrit 45% as shown in Fig. 6a, it is found that the velocity of the RBCs ranges from 8 $mm/s$ to 16.5 $mm/s$ upstream of the aneurysm region. With moving forward in the direction of the blood flow, the velocity of the RBCs decreases gradually until it reaches about 1 $mm/s$ at the center of the aneurysm. The velocity then increases with the decrease of the cross-sectional area to reach the same range of the upstream flow. With decreasing the hematocrit, the velocity of the RBCs increases in the regions up and downstream of the aneurysm, as shown in Fig. 6b and c. It reaches 22 $mm/s$ at hematocrit 20% whereas it reaches 24 $mm/s$ at hematocrit 10% as shown in Fig. 6d. Similarly, the decrease in the hematocrit slightly increases the velocity of the RBCs at the center of the aneurysm. It reaches 1.6$~mm/s$ at hematocrit 20% whereas it reaches 2.2 $mm/s$ at hematocrit 10%.

**Fig. 7:**
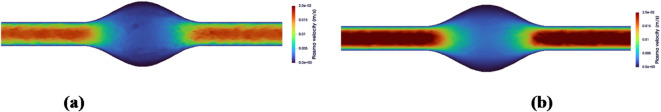
The variation of the plasma flow velocity with hematocrit variation: (a) hematocrit 45% (b) hematocrit 10%.

For all examined cases, the velocity of the RBCs decreases gradually in the divergent part of the aneurysm to reach its minimum levels at the center of the aneurysm, then it increases in the convergent part to reach its levels of the normal part of the vessel. This is due to the change in the cross-sectional area of the flow. The reduction in the velocity of the RBCs at the center of the RBCs is about 83.5% for hematocrit 45% and 87.5% for hematocrit 10%. The significant change in the velocity of the RBCs increases the aggregation of the RBCs in this region, which affects the clotting development. Furthermore, the fusiform aneurysm leads to a decrease in the plasma velocity and consequently oscillating or low WSS. The decreased WSS has several clinical implications, especially on the vessel wall integrity. For instance, it can lead to endothelial dysfunction, thrombus formation, and continued vessel dilation and weakening over time. The irregular hemodynamic forces can increase the risk of aneurysm rupture. Figure 7 depicts the effect of hematocrit variation on the contours of the plasma flow velocity for hematocrits 45% and 10%. It is found that the presence of the RBCs significantly affects the plasma velocity. With decreasing the hematocrit value, the plasma velocity increases.

### Platelet margination

**Fig. 8:**
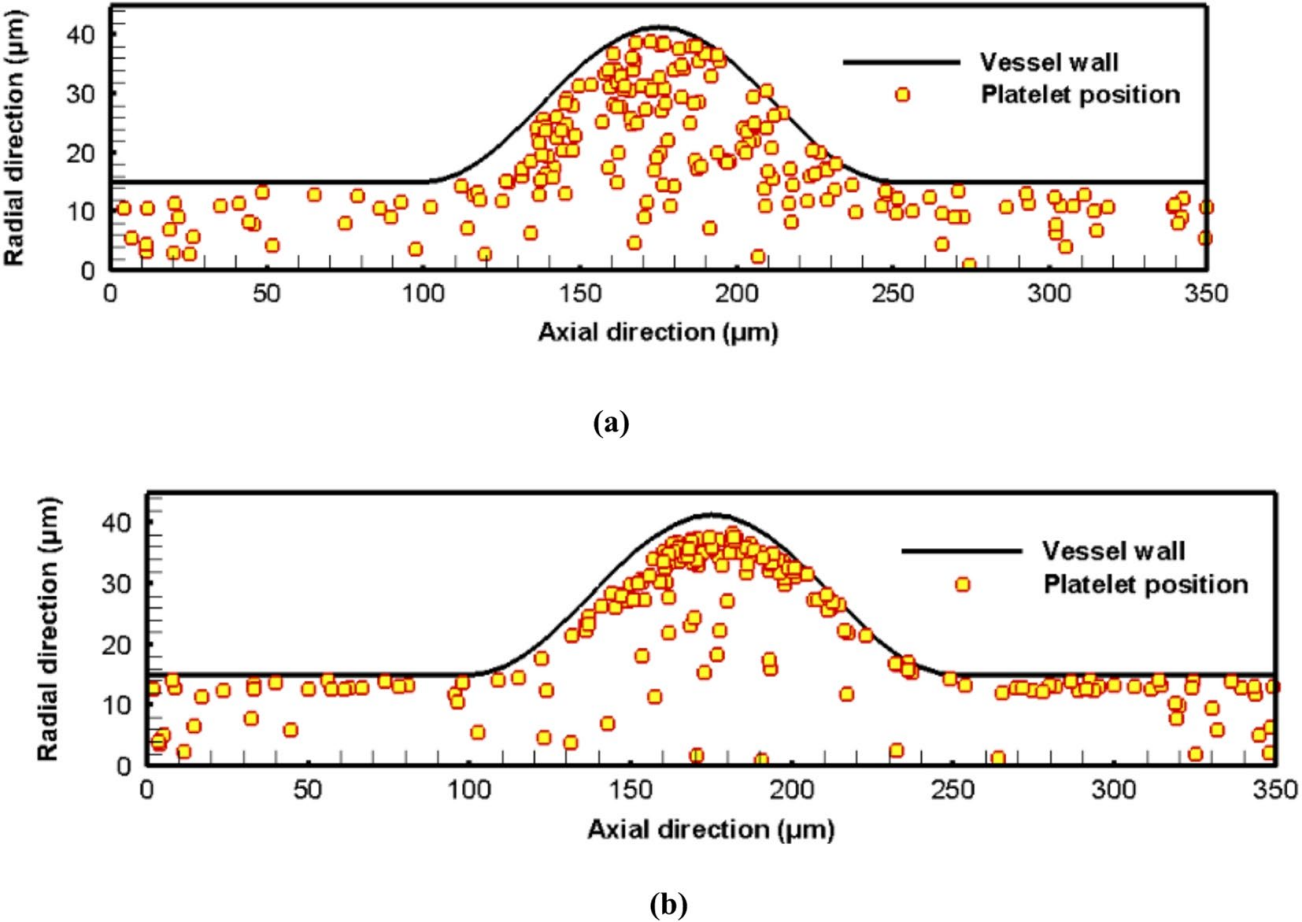
The spatial distribution of the platelets for hematocrit is 45%: (a) at the start of the simulation, (b) at the end of the simulation.

Another important feature in cellular blood flow dynamics is the increased concentration of the platelets near the vessel wall, known as platelet margination. It occurs in the presence of RBCs with sufficient hematocrit levels, more than 10%^54^. Recent experimental results have reported that the degree of platelet margination is highly affected by several factors such as the hematocrit, the channel size, and the RBCs’ deformability^55^. Platelets play a crucial role in the repair of damaged vessel walls and the process of blood clotting^56^. Additionally, it is found that platelet margination has a crucial role in the process of hemostasis and thrombosis, and hence, many studies have been performed to examine this phenomenon^57^. As a result, the platelet margination process is examined under different hematocrit levels. While the performed simulations depended on a validated code, HemoCell, that was developed for cellular blood flow simulations, the obtained results are also consistent with other published studies, which enhances the results’ validity. For instance, Chang et al.^27^ simulated the flow of the RBCs and platelets in straight vessels. Their results predicted the margination of the platelets towards the vessel wall for different hematocrit levels. Figure 8a depicts the position of the platelets in the vessel as well as the fusiform aneurysm at the start of the simulation, while Fig. 8b shows the position of the platelets at the end of the simulation for Reynolds number 0.5 and hematocrit 45%. Comparing the results obtained for the platelet positions at the start and the end of the simulation, it is found that the migration of the platelet is confirmed, where the platelets move towards the vessel wall, especially in the aneurysm zone.

**Fig. 9:**
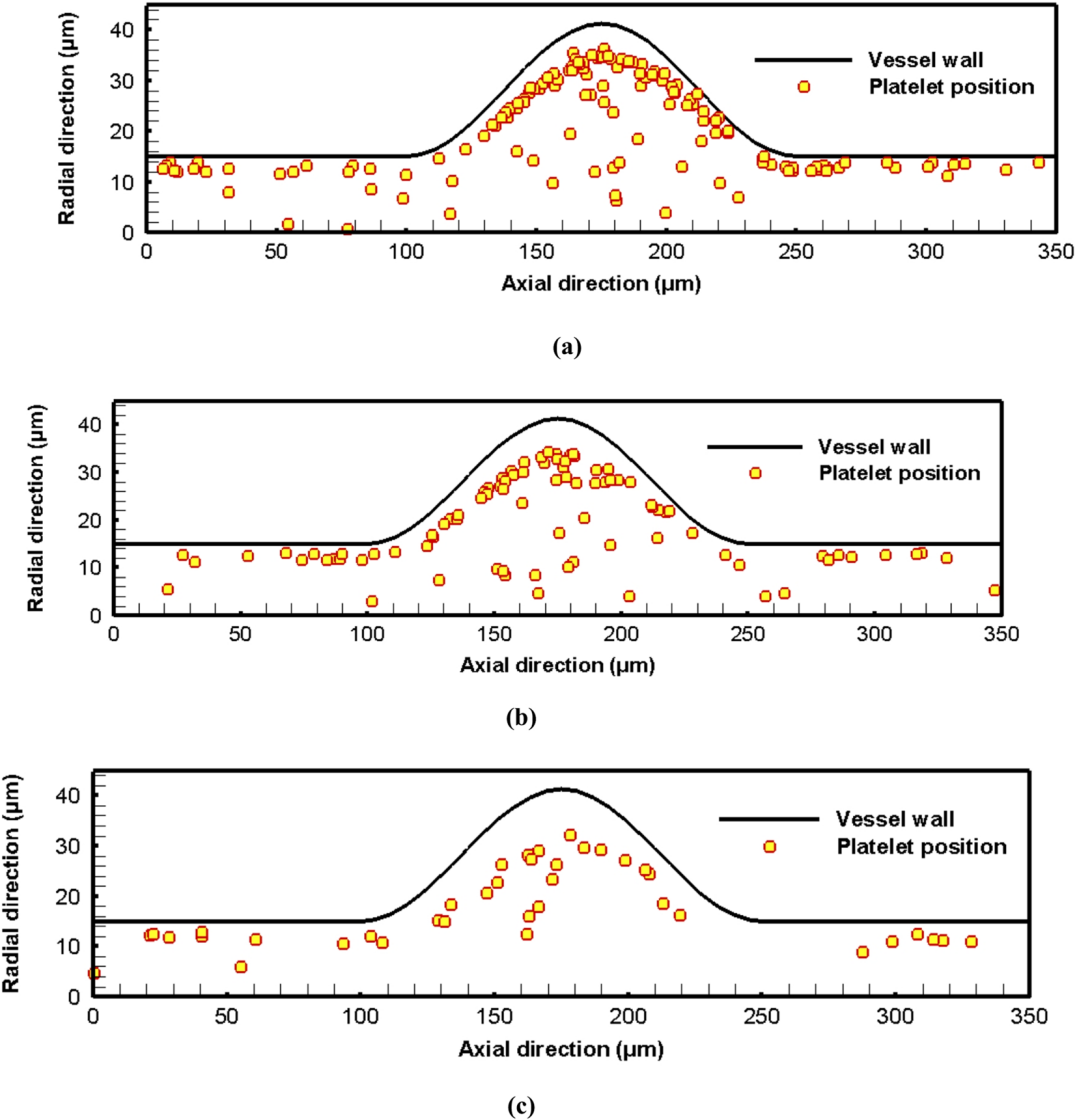
The spatial positions for the platelets for different hematocrit levels: (a) hematocrit 45%, (b) hematocrit 30%, (c) hematocrit 20%, and (d) hematocrit 10%.

**Fig. 10:**
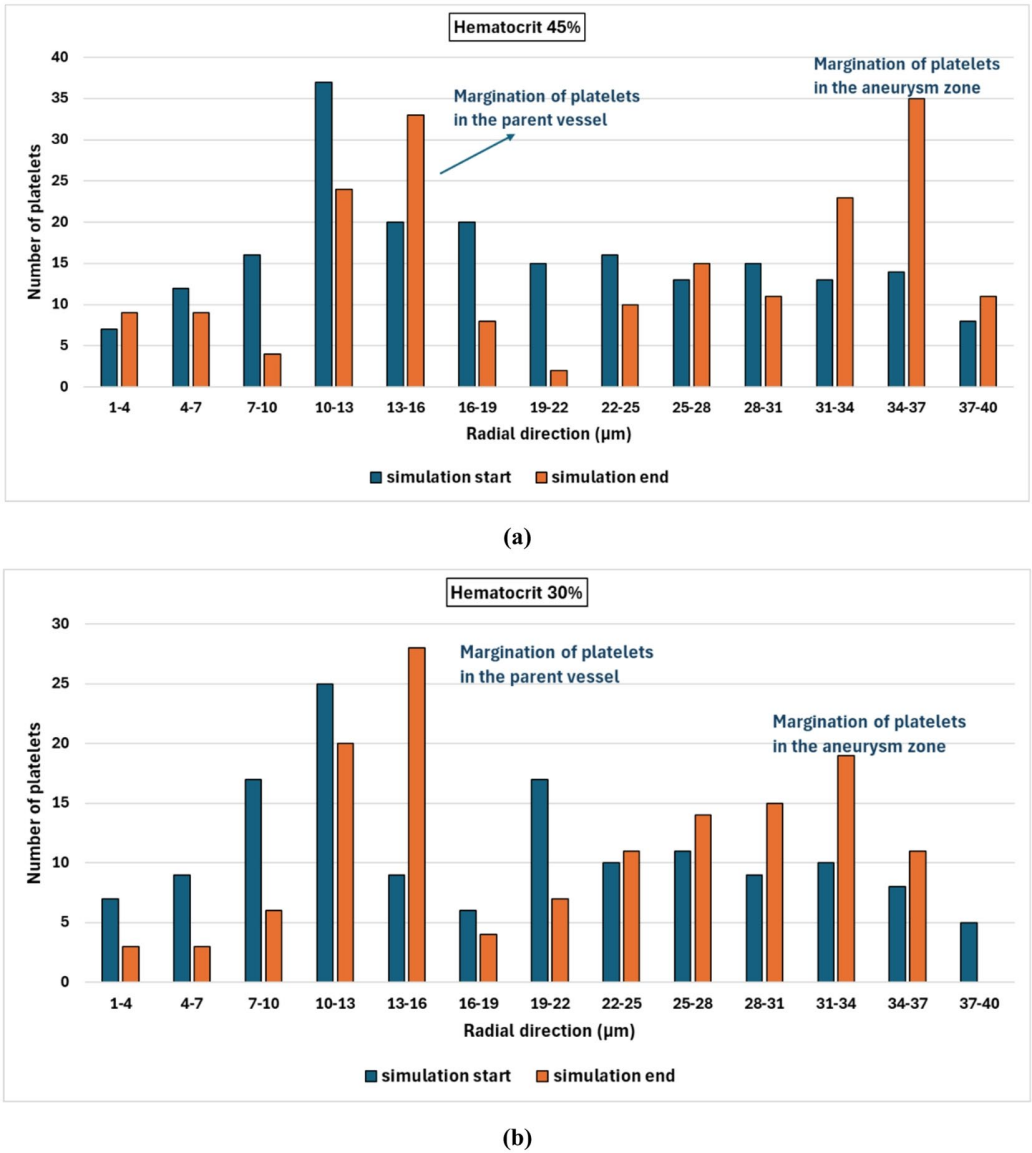

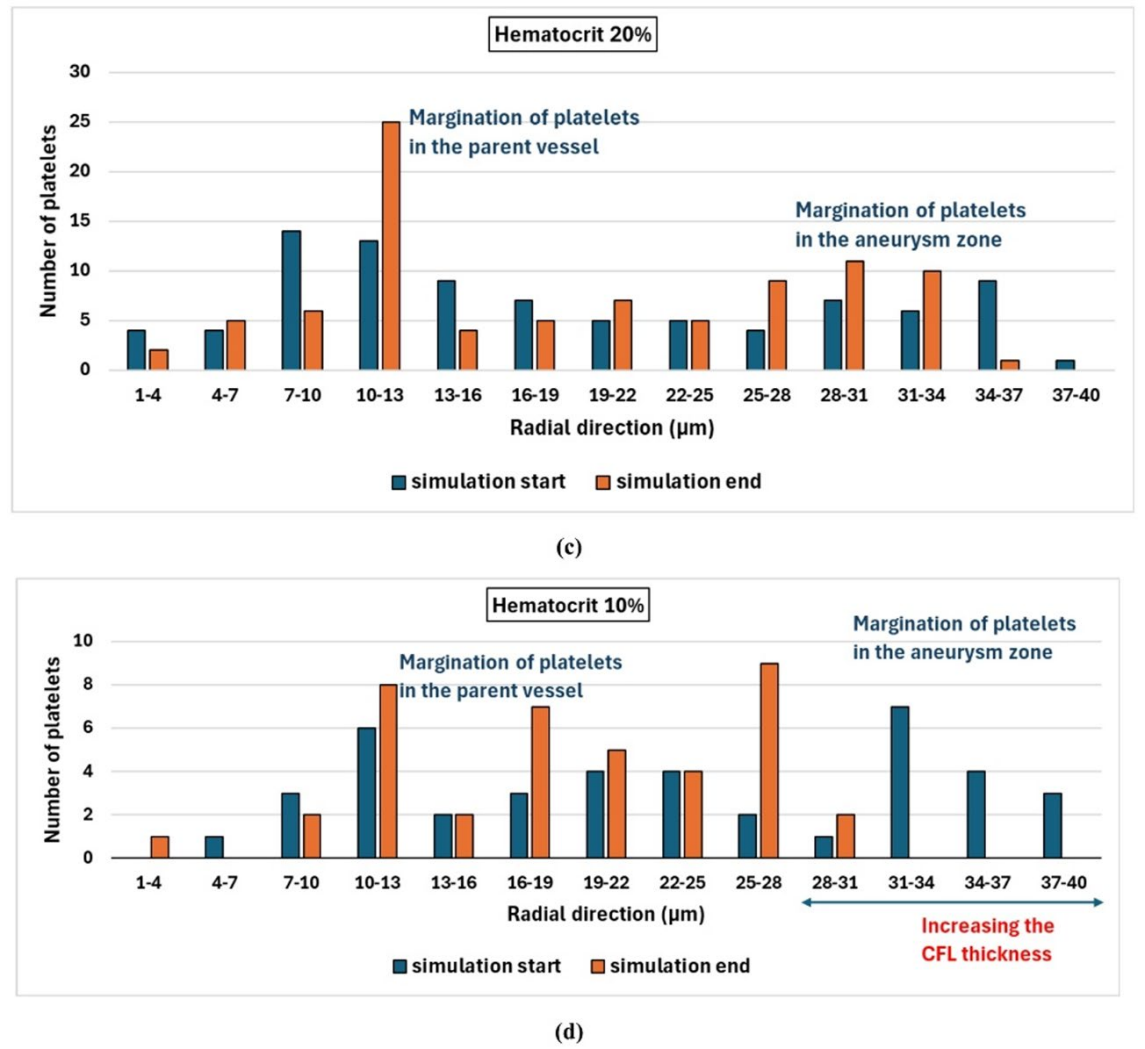
Histograms for the radial positions of platelets’ margination at the start and the end of the simulations for different hematocrit levels: (a) hematocrit 45%, (b) hematocrit 30%, (c) hematocrit 20%, and (d) hematocrit 10%.

To quantify the effect of the hematocrit level on the migration process, the proportion of migrated platelets is calculated for different hematocrit levels. The radial positions for the platelets for different hematocrits are shown in histograms in Fig. 10.

It is found that most of the platelets concentrate near the vessel wall regardless of the hematocrit level. However, the distribution of the platelets inside the aneurysm zone is highly affected by the hematocrit level. For hematocrit 45%, the maximum radial position for the platelet is about 40$~\mu m$ which means that about 1.27$~\mu m$ of a layer thickness is free of platelets as shown in Fig. 10a. With decreasing the hematocrit level, it is found that the thickness of this layer increases to reach about 6 $\mu m$ at hematocrit 10% as shown in Fig. 10d. It is clear that the RBCs induce the movement of the platelets toward the vessel wall. This result is consistent with experimental results as reported by Sugihara-Seki et al.^55^. They stated that the degree of margination is affected by the hematocrit level. As the fusiform aneurysm leads to disturbed flow with recirculation zones and low WSS, the platelet dynamics are highly affected. The fusiform aneurysm promotes platelet adhesion and activation, which increases the concentration of the platelets near the vessel wall compared with the parent vessel^58^. The Platelet activation and the thrombus formation contribute to the weakening of the vessel wall, and hence the progression of the aneurysm may lead to wall rupture.

### Cell-free layer thickness

**Fig. 11:**
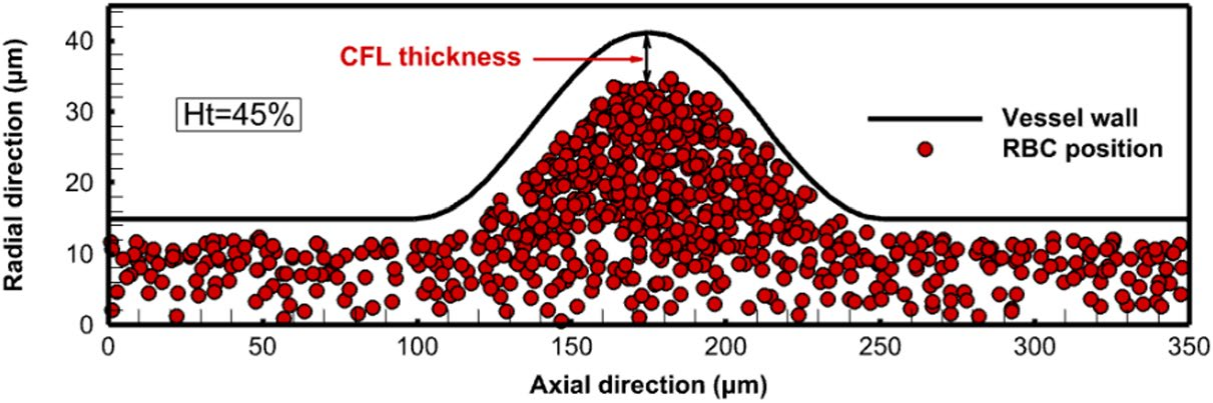
The spatial distribution of the RBCs through the vessel at the end of the simulation (hematocrit 45%).

**Fig. 12:**
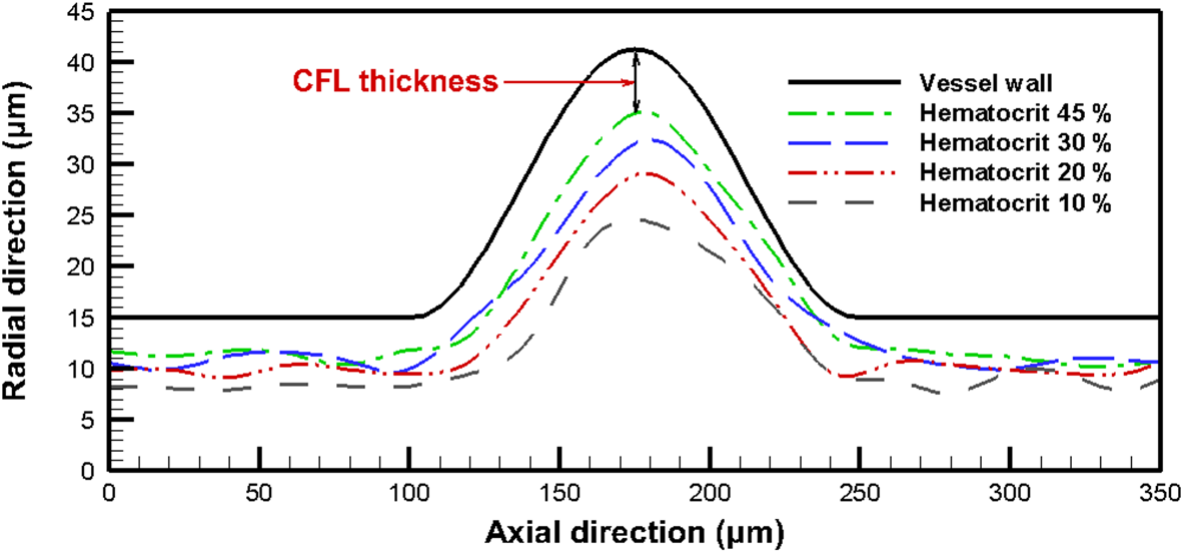
The variation of the CFL thickness with hematocrit variation.

**Fig. 13:**
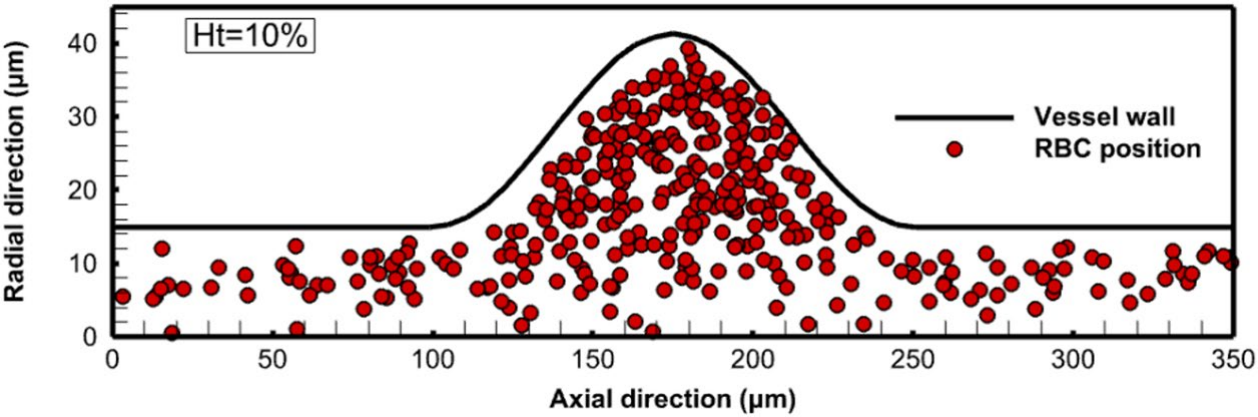
The spatial distribution of RBCs through the vessel at the start of the simulation (hematocrit 10%).

In the current study, an important feature related to the migration of the RBCs, the CFL, is examined. The formation of the CFL is due to the migration of the RBCs towards the flow center. This type of movement is induced by the tank treading motion of the deformable cell membrane^59^. The CFL thickness is affected by some factors, such as the hematocrit value, the flow rate, and the tube diameter. In the present work, the CFL thickness is approximated as the distance from the vessel wall to the center of the outer flowing RBCs as indicated in Fig. 11. The variation of the CFL thickness with the hematocrit level is considered as shown in Fig. 12. The CFL thickness is calculated along the axial direction, and the results are obtained for Reynolds number 0.5. In the normal part of the vessel, the CFL thickness at hematocrit 45% is about 3.68 $\mu m$, and it increases with the decrease of the hematocrit level to reach about 6.96 $\mu m$ at hematocrit 10%. The effect of the hematocrit on the CFL thickness appears clearly in the aneurysm zone, where the velocity of the RBCs decreases, and the aggregation increases. Furthermore, it is noted that the maximum CFL thickness occurs at the center of the aneurysm. At the center of the aneurysm, the CFL thickness reaches 6.22 $\mu m$ at hematocrit 45%. With a decrease in the hematocrit level to 10%, the CFL thickness is about 16.67 $\mu m$ compared with a thickness of 2.03 $\mu m$ at the start of the simulation, as shown in Fig. 13. The fusiform aneurysm, as shown in Fig. 12, has a remarkable effect on the CFL thickness. It is found that the CFL thickness increases in the aneurysm zone. This increase is due to several hemodynamic factors. For example, the circumferential dilation of the fusiform aneurysm decreases the plasma velocity and hence decreases the shear rate in this region. As a result, the lift forces that tend to push the RBCs towards the wall decrease, which keeps the RBCs concentrated in the core of the vessel and increases the CFL thickness. Furthermore, the dilated geometry of the aneurysm zone allows for the formation of vortices, and hence these flow structures trap RBCs towards the centerline of the vessel, and hence increase the CFL thickness.

Another interesting thing is that the CFL thickness exhibits an asymmetrical trend up- and downstream of the aneurysm zone. It is found that the CFL thickness upstream of the aneurysm is thicker than the CFL thickness in the convergent part of the aneurysm. This is due to the increased accumulation of the RBCs in this region. Furthermore, the convergent part of the fusiform aneurysm increases the velocity of the plasma and the RBCs. This increases the shear rate in this region. The increased shear rate increases the lift forces that push the RBCs towards the wall and decreases the CFL thickness. The increase in the CFL thickness has several clinical implications such as reduced blood viscosity and hence the flow resistance. Additionally, increasing the CFL thickness decreases the risk of endothelial injury resulting from the movement of the RBCs near the vessel wall. The obtained results are consistent with those obtained by Fedosov et al.^26^. In their study, the CFL thickness was computed for different hematocrit levels. They found that with decreasing the hematocrit level, the CFL thickness increased, which greatly matches the obtained results. A similar conclusion is obtained for a simulation of the cellular blood flow in a micro-vessel with a stenosis performed by Xiao et al.^24^. In their study, they found that the blood flow exhibited asymmetry in the CFL thickness along the flow direction, where the aggregation of the RBCs before the stenosis reduced the CFL thickness.

### Plasma flow in the fusiform aneurysm

To complete the picture of the cellular blood flow in micro-fusiform aneurysms, the effect of hematocrit variation on the plasma flow is examined. The plasma flow velocity as well as the WSS are calculated at hematocrits 45%, 30%, 20%, and 10%. The results are obtained for Reynolds number 0.5.

#### Plasma flow velocity

**Fig. 14:**
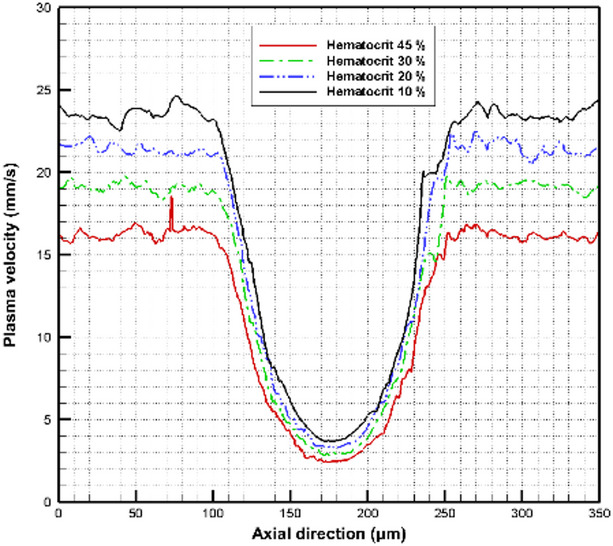
The variation of the plasma flow velocity in the axial direction with hematocrit variation.

Figure 14 depicts the plasma velocity in the axial direction for different hematocrit values. In the normal part of the vessel, the plasma velocity is about $16~mm/s$ at hematocrit 45% whereas it reaches $23~mm/s$ at hematocrit 10%. The plasma velocity decreases significantly in the aneurysm zone. For hematocrit 45%, the plasma velocity is about $2.6~mm/s$ while it reaches about $3.9~mm/s$ at hematocrit 10%.

**Fig. 15:**
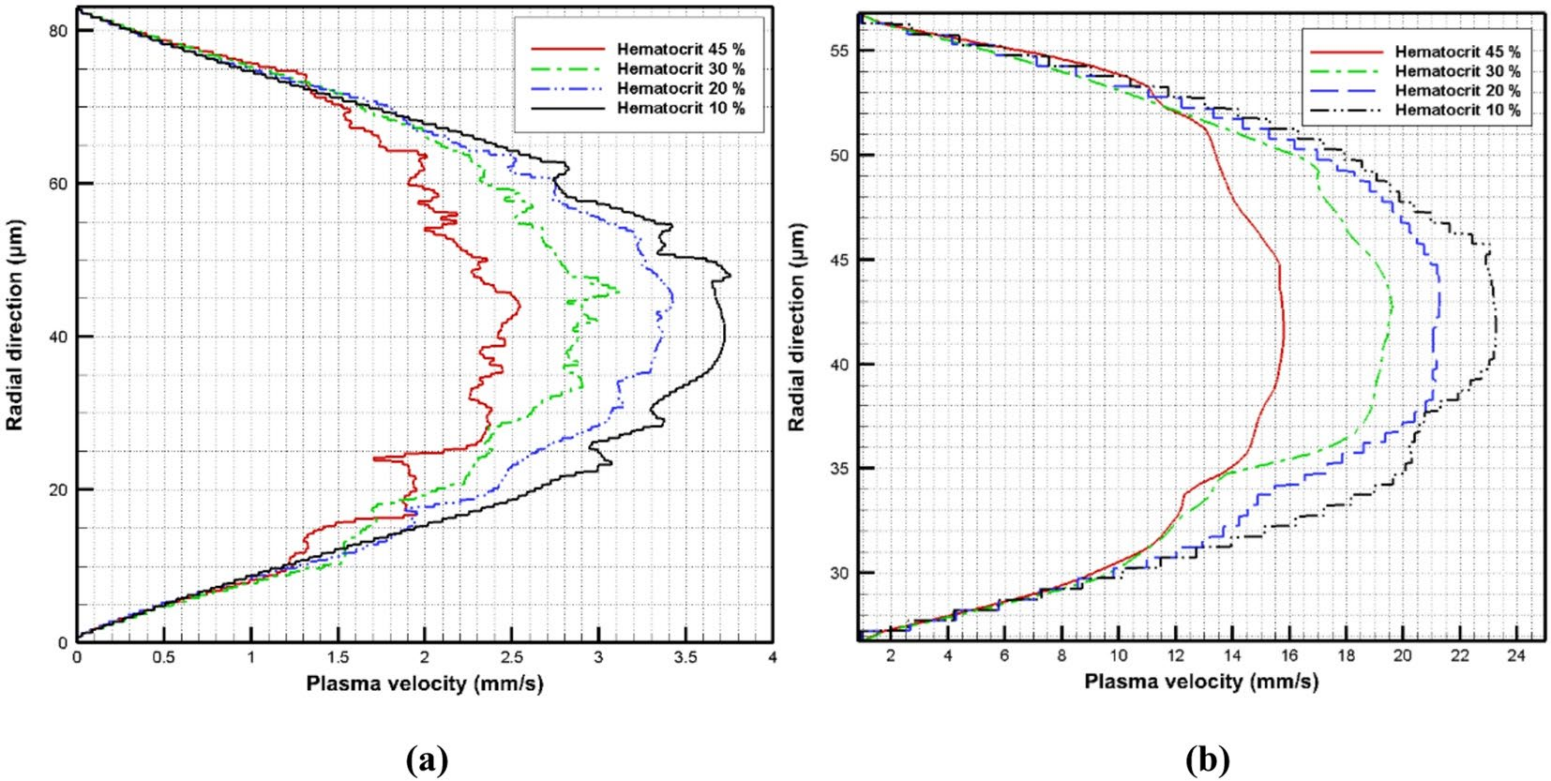
The variation of the plasma flow velocity in the radial direction with hematocrit variation: (a) at the center of the aneurysm and (b) near the vessel exit.

The effect of hematocrit variation on the plasma velocity is also indicated by calculating the plasma velocity in the radial direction at two different positions in the vessel as shown in Fig. 15. The plasma velocity is calculated first at the center of the aneurysm as shown in Fig. 15a. It is found that there is no velocity variation near the vessel wall while the plasma velocity changes clearly at the core of the vessel with the variation of the hematocrit. The plasma velocity is also calculated far from the aneurysm zone near the vessel exit, as shown in Fig. 15b. The plasma velocities are higher than the velocities in the aneurysm zone.

#### Wall shear stress

The LBM allows for calculating the viscous stress tensor from the lattice as follows^50^:

23 $${\varvec{\sigma}_{x,y,z}}= - \left( {1 - \frac{{\delta t}}{{2\tau }}} \right){\mathbf{\Pi }}_{{x,y,z}}^{{\left( 1 \right)}}$$

where $\delta t$ is the time step, $\tau$ is the relaxation time and ${\text{\varvec{\Pi}}}_{{x,y,z}}^{{\left( 1 \right)}}$ is the perturbation momentum. The WSS can be calculated from the dot product of the viscous stress tensor with the surface normal vector$~{\varvec{n}}$ of the vessel wall as follows:

24 $${\varvec{\tau}_w}={\varvec{\sigma}_{x,y,z}} \cdot {\varvec{n}}$$

**Fig. 16:**
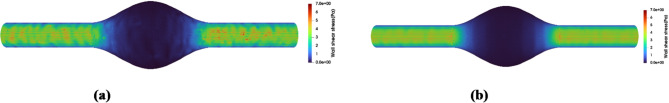
The contours of the WSS with hematocrit variation: (a) hematocrit 45% and (b) hematocrit 10%.

**Fig. 17:**
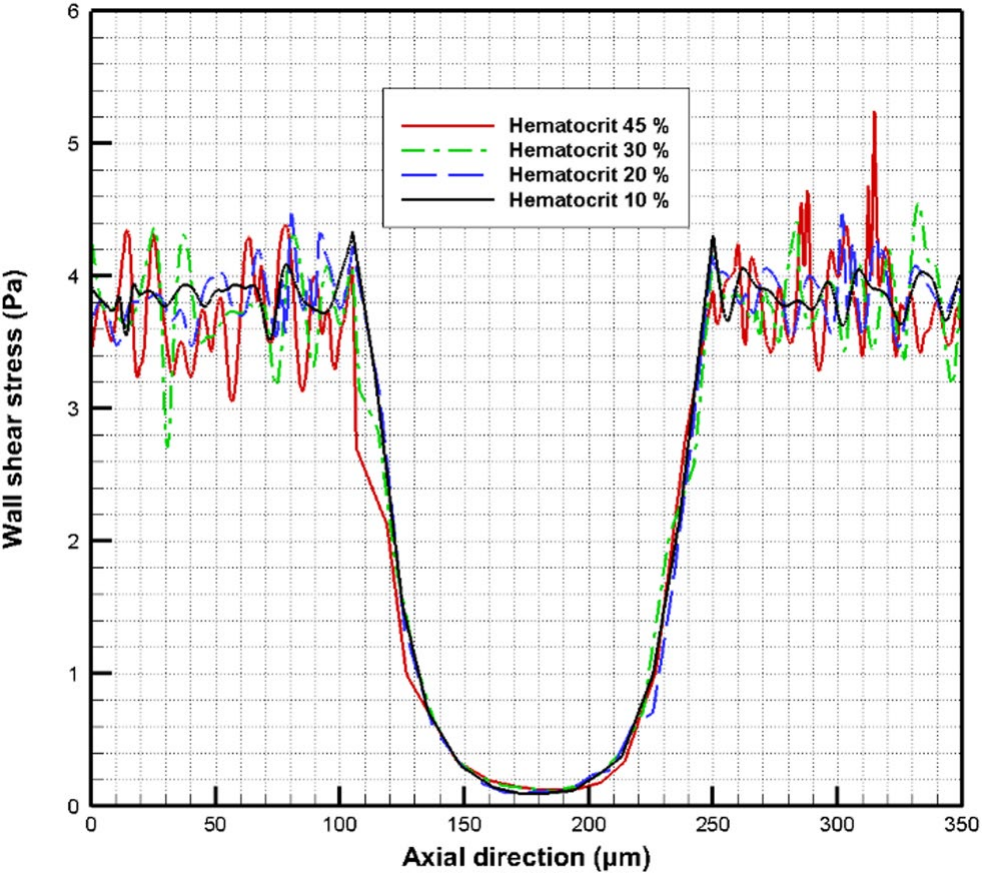
The variation of the WSS with hematocrit variation.

The effect of hematocrit variation on the WSS is considered for hematocrits 45%, 30%, 20%, and 10%. Figure 16 shows the WSS contours for hematocrits at 45% and 10%. The maximum WSS occurs in the normal part of the vessel, whereas the WSS decreases significantly in the aneurysm zone. Comparing the results obtained in Fig. 16, it is clear that the WSS at the hematocrit level of 45% is slightly more than the WSS at the hematocrit level of 10%. The variation of the WSS can be indicated clearly, as shown in Fig. 17.

## Conclusion

Examining cellular blood flow dynamics based on numerical simulations at the microscale level is important to have a better understanding of several diseases, such as diabetic retinopathy. As a result, the current work’s objective is to perform numerical simulations of cellular blood flow dynamics in micro-vessels with a fusiform aneurysm that are not considered in the literature. The performed simulations are based on a validated code, HemoCell, developed for the simulation of high-density suspensions of blood cells. The LBM coupled with the IBM is used for plasma-cell interaction. In the present study, some key features in cellular blood flow dynamics provide more insights into the nature of blood flow inside micro-aneurysms. The RBCs’ migration and their velocities, the platelets’ margination, the CFL thickness, and the plasma flow are considered. Both RBCs’ migration and platelets’ margination are confirmed under different hematocrit levels which proves the efficiency of the proposed code in simulating such flows. The obtained results show that the migration of the RBCs is strongly affected by the hematocrit value, and hence the CFL thickness which agrees with published works. It is noted that the maximum CFL thickness occurs at the center of the aneurysm. As a result, the apparent viscosity of blood in this region decreases significantly which strongly affects the WSS, and hence the endothelial cell function of vessel walls. An asymmetrical CFL is predicted in the aneurysm zone where the CFL thickness downstream of the aneurysm is thinner than the CFL thickness in other regions which induces the fluctuation of the WSS. Furthermore, it is found that the accumulation of the RBCs occurs at the center of the aneurysm where the velocity of the RBCs is reduced as well as the plasma velocity. For the margination of the platelets, the hematocrit levels affect the concentration of the platelets near the vessel wall where the maximum cell-free layer thickness occurs at hematocrit 10%. While the hematocrit value affects plasma velocity, the WSS is slightly affected. In the end, the current findings prove that studying the cellular blood flow at the cellular level provides a better understanding of prominent features in micro-aneurysms that cannot be explored by the conventional continuum analysis.

## Supplementary Information

Below is the link to the electronic supplementary material.

Supplementary Material 1

## Data Availability

Data sets generated during the current study are available from the corresponding author on reasonable request.
