# Supplementary material for: Numerical simulation of red blood cells migration and platelets margination for blood flow in micro-vessels with fusiform aneurysms
Source: Sci Rep. 2025 Oct 27;15:37350. doi: 10.1038/s41598-025-22429-w (PMC12559442; doi:10.1038/s41598-025-22429-w)
Supplement: Supplementary file 1 — Supplementary Material 1 [file 41598_2025_22429_MOESM1_ESM.docx]

**Appendices**

**Appendix I The mechanical model for the RBCs and platelets**

In HemoCell, the cell membrane is descritized using a triangular mesh as shown in **Fig. AI. 1**. The triangular mesh is considered a spring network which is used to approximate the real spectrin network of the RBCs. For the RBCs membrane, the spring network consists of 642 vertices, 1920 edges and 1280 faces, whereas the platelet membrane consists of 66 vertices and 128 triangular faces. The deformation of the RBC membrane is modeled using a set of four applied forces. [33]. The set of applied forces is described as follows:

1. The first force is the link force, which acts along the links between the vertices and represents the stretch and the compression occurring in the spectrin network. The link force is mathematically expressed as follows:

$\vec{F}_{link}=-\frac{k_{l}dL}{P}\left[ 1+\frac{1}{{\tau_{l}}^{2}-{dL}^{2}} \right],$ (AI.1)

where$dL$ is the normal strain;$L_{0}$ is the equilibrium length; $k_{l}$ is the link stretch modulus;$L_{i}$ is the actual length; $P$ is the persistence length; and $\tau_{l}$ is the relative expansion ratio for length at $\tau_{l}=$ 3.0.

1. The second force is the bending force, which acts between two adjacent faces and acts on the normal direction of each face. The bending force can be described mathematically as follows:

$\vec{F}_{bend}=-\frac{k_{b}d\theta}{L_{0}}\left[ 1+\frac{1}{{\tau_{b}}^{2}-{d\theta}^{2}} \right],$ (AI.2)

where $\theta_{i}$ and $\theta_{0}$ are the instantaneous and equilibrium angles between two adjacent faces, respectively. $d\theta=\theta_{i}-\theta_{0}$, $\tau_{b}$ is the limiting angle, and$k_{b}$ is the bending modulus.

1. The third force is the local surface conservation force. This force represents the surface response to both stretching and compression. The mathematical formulation of the force is similar to the link force:

$\vec{F}_{area}=-\frac{k_{a}dA}{L_{0}}\left[ 1+\frac{1}{{\tau_{a}}^{2}-{dA}^{2}} \right],$ (AI.3)

where$dA=\frac{A_{i}-A_{0}}{A_{0}}$;$A_{i}$, and $A_{0}$ are the actual and equilibrium surface areas; $k_{a}$ is the local area constraint modulus; and the relative expansion ratio for the area $\tau_{a}=0.3$.

1. The fourth force is the volume conservation force. The volume conservation force is described mathematically as follows:

$\vec{F}_{volume}=-\frac{k_{v}dV}{L_{0}}\left[ \frac{1}{{\tau_{v}}^{2}-{dV}^{2}} \right],$ (AI.4)

where $dV=\frac{V_{i}-V_{0}}{V_{0}}$;$V_{i}$, and $V_{0}$ are the current and equilibrium volume of the cell; $k_{v}$ is the volume constraint modulus; and the relative expansion ratio for volume $\tau_{v}=$0.01.

**
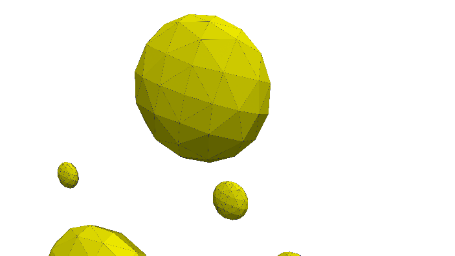

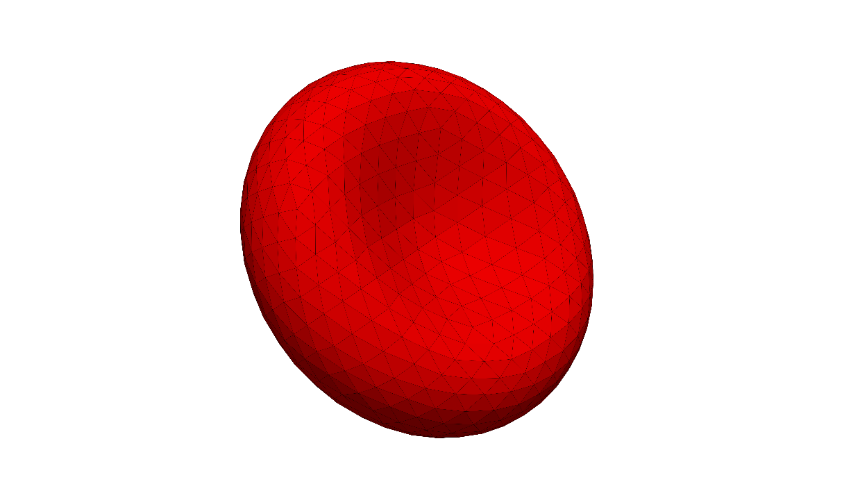
**

1. **(b)**

**Figure AI. 1: The triangular mesh of blood cells: (a) the RBC, (b) the platelet.**

**Appendix II The numerical values of the parameters used in the current study**

In the current study, healthy blood cells are considered, and hence, certain values of the parameters are chosen to fit the healthy cell properties are summarized in the following tables:

**Table AII. 1: The numerical values of the parameters used in the simulation** [7]**.**

| Symbol | Numerical value |
| --- | --- |
| $L_{0}$ | $0.5$ |
| $\tau_{b}$ | $\frac{\pi}{2}$ |
| P | $7.5$ |
| $E_{s}$ | 27.82 |

**Table AII. 2: The numerical values of the free parameters for both the RBC and the platelet.**

| symbol | Red blood cells | Platelets |
| --- | --- | --- |
| $\boldsymbol{k}_{\boldsymbol{l}}$ | 15 | 25 |
| $\boldsymbol{k}_{\boldsymbol{b}}$ | 80 | 250 |
| $\boldsymbol{k}_{\boldsymbol{a}}$ | 5 | 8 |
| $\boldsymbol{k}_{\boldsymbol{v}}$ | 20 | 100 |

**Appendix III The Lattice Boltzmann Method (LBM)**

The LBM has become a strong alternative to computational fluid dynamics numerical methods [42]. One of the most important benefits of LBM is its validity for parallel computations. It is based on the kinetic theory of gases, in which the Boltzmann transport equation is defined as follows:

$\frac{\partial f}{\partial t}+\vec{c}\cdot\vec{\nabla}f=\Omega,$ (AIII.1)

where $\vec{c}$ is the particle velocity, $f$ is the particle distribution function,and $\Omega$ is the collision operator. The discretized equation is described as follows:

$f_{i}\left( \vec{x}+\vec{c}_{i}\Delta t,t+\Delta t \right)=f_{i}\left( \vec{x},t \right)+\Omega_{i}\left( f \right)+\Delta t\vec{F}_{i}\left( \vec{x},t \right),$ (AIII.2)

the discretized collision operator approximated by the BGK model [53] with a single relaxation time and external forces can be written as follows

$\Omega_{i}\left( f \right)=-\frac{f_{i}\left( \vec{x},t \right)-{f_{i}}^{eq}\left( \vec{x},t \right)}{\tau},$ (AIII.3)

where ${f_{i}}^{eq}$ is the equilibrium distribution function, $\tau$ is the single relaxation time, $F_{i}\left( \vec{x},t \right)$ is the external force term and $\vec{f}$ is the body force density:

$\vec{F}_{i}\left( \vec{x},t \right)=\left( 1-\frac{1}{2\tau} \right)\omega_{i}\left( \frac{\vec{c}_{i}-\vec{u}}{{c_{s}}^{2}}+\frac{\vec{c}_{i}\cdot\vec{u}}{{c_{s}}^{4}}\vec{c}_{i} \right)\cdot\vec{f},$ (AIII.4)

${f_{i}}^{eq}\left( \vec{x},t \right)=\omega_{i}\rho\left( 1+\frac{\vec{c}_{i}\cdot\vec{u}}{{c_{s}}^{2}}+\frac{\left( \vec{c}_{i}\cdot\vec{u} \right)^{2}}{2{c_{s}}^{4}}-\frac{\vec{u}\cdot\vec{u}}{2{c_{s}}^{2}} \right),$ (AIII.5)

$\rho=\sum_{i} f_{i},$ (AIII.6)

$\rho\vec{u}=\sum_{i} f_{i}\vec{c}_{i}+\frac{\Delta t}{2}\vec{f},$ (AIII.7)

where $c_{s}=\frac{1}{\sqrt{3}}\frac{\Delta x}{\Delta t}$ is the lattice speed of sound and $\omega_{i}$ are the lattice weight coefficients. In Hemocell, a D3Q19 lattice model is proposed [33]. The lattice weight coefficients $\omega_{i}$ can be defined as follows:

$\omega_{i}=\left\{ \begin{matrix} \frac{1}{3} for i=0 \\ \frac{1}{18} for i=1-6 \\ \frac{1}{36} for i=7-18 \end{matrix} \right..$ (AIII.8)

**Appendix IV The Immersed Boundary Method (IBM)**

The interaction between the immersed elastic membrane and the fluid can be captured by considering the membrane forces as local fluid forces [54]. The fluid domain is discretized using a fixed Eulerian mesh, whereas the immersed boundary is represented using the Lagrangian coordinates [55]. The interaction between the surrounding plasma and the elastic membrane can be modeled by obtaining the relationship between the Lagrangian and the Eulerian forces and velocities. The mathematical formulation of IBM is briefly introduced as follows:

The relation between the local Lagrangian force $\mathbf{F}$ and the Eulerian$\mathbf{f}$ can be obtained from the following relation [8]

$\vec{\mathbf{f}}=\int_{\Gamma} \mathbf{F}\delta\left[ \boldsymbol{x}-\boldsymbol{X} \right]ds,$ (AIV.1)

where $\delta$ is the Dirac delta function in 3D and $\mathbf{X}$ is the position vector for the Lagrangian coordinate system. In IBM, a no-slip boundary condition is imposed at the surface of the immersed boundary [56], that is

$\boldsymbol{U}\left( s,t \right)=\boldsymbol{u}\left( \mathbf{X}\left( s,t \right),t \right)=\frac{\partial\boldsymbol{X}(s,t)}{\partial t},$ (AIV.2)

where $\boldsymbol{U}\left( s,t \right)$ is the velocity of the Lagrangian nodes, whereas $\boldsymbol{u}\left( \boldsymbol{x},t \right)$ is the velocity of the Eulerian fluid nodes. The velocity of the Lagrangian nodes can be updated by interpolating the velocity of the Eulerian nodes as follows:

$\boldsymbol{U}\left( \boldsymbol{s},t \right)=\int_{\Omega} \boldsymbol{u}(\boldsymbol{x},t)\delta\left[ \boldsymbol{x}-\mathbf{X}(s,t) \right]dx.$ (AIV.3)

The updated positions of the Lagrangian nodes can be obtained as follows:

$\mathbf{X}\left( s,t+\Delta t \right)=\mathbf{X}\left( s,t \right)+\boldsymbol{U}\left( s,t \right)\Delta t.$ (AIV.4)

The solution algorithm for the RBCs flow in plasma and the combination of the LBM-IBM can be summarized in the following figure:

**
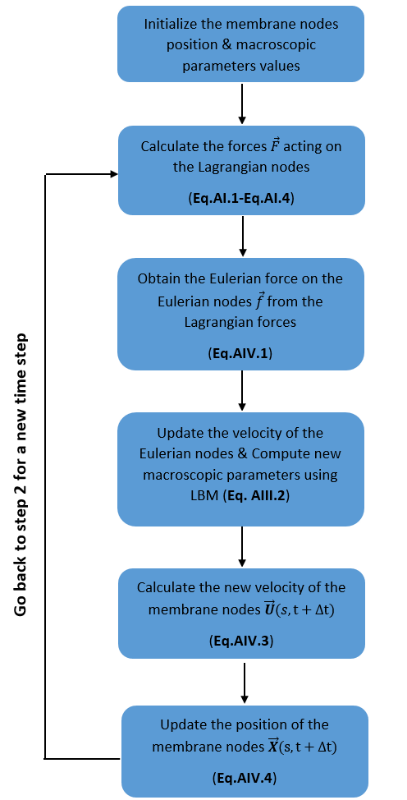

Figure AIV. 1: The solution algorithm for the interaction between the cell deformation and the surrounding plasma using LBM-IBM.**
